# ACTIVITY SELECTION AND ENGAGEMENT IN OLD AGE: MOTIVATIONAL AND GOAL-BASED INFLUENCES

**DOI:** 10.1093/geroni/igz038.2991

**Published:** 2019-11-08

**Authors:** Thomas M Hess, Christopher Hertzog

**Affiliations:** 1 North Carolina State University, Raleigh, North Carolina, United States; 2 Georgia Institute of Technology, Atlanta, Georgia, United States

## Abstract

Research from a variety of perspectives has emphasized the central role played by activity in supporting a variety of positive outcomes in later life. For example, participation in activities that place demands on personal resources has been shown to be beneficial in promoting brain, cognitive, and physical health. From another perspective, older adults may also engage in certain activities to promote specific outcomes (e.g., emotional) in service of psychological well-being. Such findings highlight the adaptive significance of activity selection and engagement processes. Using a variety of approaches, the presentations in this symposium focus specifically on goal-based and motivational factors that may facilitate or impede such processes. Moored and colleagues examine adaptive characteristics—including motivational ones—of individuals whose activity patterns are protective against dementia. Using longitudinal data from the Health and Retirement Study, Lothary and colleagues explore the degree to which intrinsic motivation to engage cognitive resources mediates the effect of personal resources (e.g., physical and emotional health) on participation in challenging everyday activities. Growney and colleagues present research demonstrating that subjective perceptions of difficulty affect decisions to engage in challenging activities, but that such perceptions may reflect biases associated with negative aging attitudes as opposed to actual effort expenditure. Finally, Lind and Isaacowitz examine selection associated with affective aspects of the activities, finding that both middle-aged and older adults exhibited similar biases toward positive activities in congruence with emotion-regulation goals, though age differences were observed in non-affective aspects of the activities. The discussion by Hertzog will highlight common themes.

